# Supplementary material for: SARS-CoV-2 infection results in immune responses in the respiratory tract and peripheral blood that suggest mechanisms of disease severity
Source: Nat Commun. 2022 May 19;13:2774. doi: 10.1038/s41467-022-30088-y (PMC9120039; doi:10.1038/s41467-022-30088-y)
Supplement: Supplementary file 1 — Supplementary Information [file 41467_2022_30088_MOESM1_ESM.pdf]

SUPPLEMENTARY INFORMATION

SARS-CoV-2 infection results in immune responses in the respiratory tract and peripheral blood that suggest mechanisms of disease severity

Wuji Zhang[1], Brendon Y. Chua[1,2], Kevin J. Selva[1], Lukasz Kedzierski[1,3], Thomas M. Ashhurst[4,5], Ebene R. Haycroft[1], Suzanne K. Shoffner-Beck[6], Luca Hensen[1], David F. Boyd[7], Fiona James[8], Effie Mouhtouris[8], Jason C. Kwong[1,8], Kyra Y. L. Chua[8], George Drewett[8], Ana Copaescu[8], Julie E. Dobson[9], Louise C. Rowntree[1], Jennifer R. Habel[1], Lilith F. Allen[1], Hui-Fern Koay[1], Jessica A. Neil[1], Matthew Gartner[1], Christina Y. Lee[6], Patiyan Andersson[10], Sadid F. Khan[11], Luke Blakeway[11], Jessica Wisniewski[11], James H. McMahon[11,12], Erica E Vine[13,14,15], Anthony L Cunningham[13,15], Jennifer Audsley[16], Irani Thevarajan[16,17], Torsten Seemann[10], Norelle L. Sherry[8,10], Fatima Amanat[18,19], Florian Krammer[18], Sarah L. Londrigan[1], Linda M. Wakim[1], Nicholas J.C. King[4,5,14,20-22], Dale I. Godfrey[1], Laura K. Mackay[1], Paul G. Thomas[7], Suellen Nicholson[23], Kelly B. Arnold[6], Amy W. Chung[1], Natasha E. Holmes[8,24-26], Olivia C. Smibert[8,27,28], Jason A. Trubiano[26-29#], Claire L. Gordon[1,8#], Thi H.O. Nguyen[1#] and Katherine Kedzierska[1,2#]

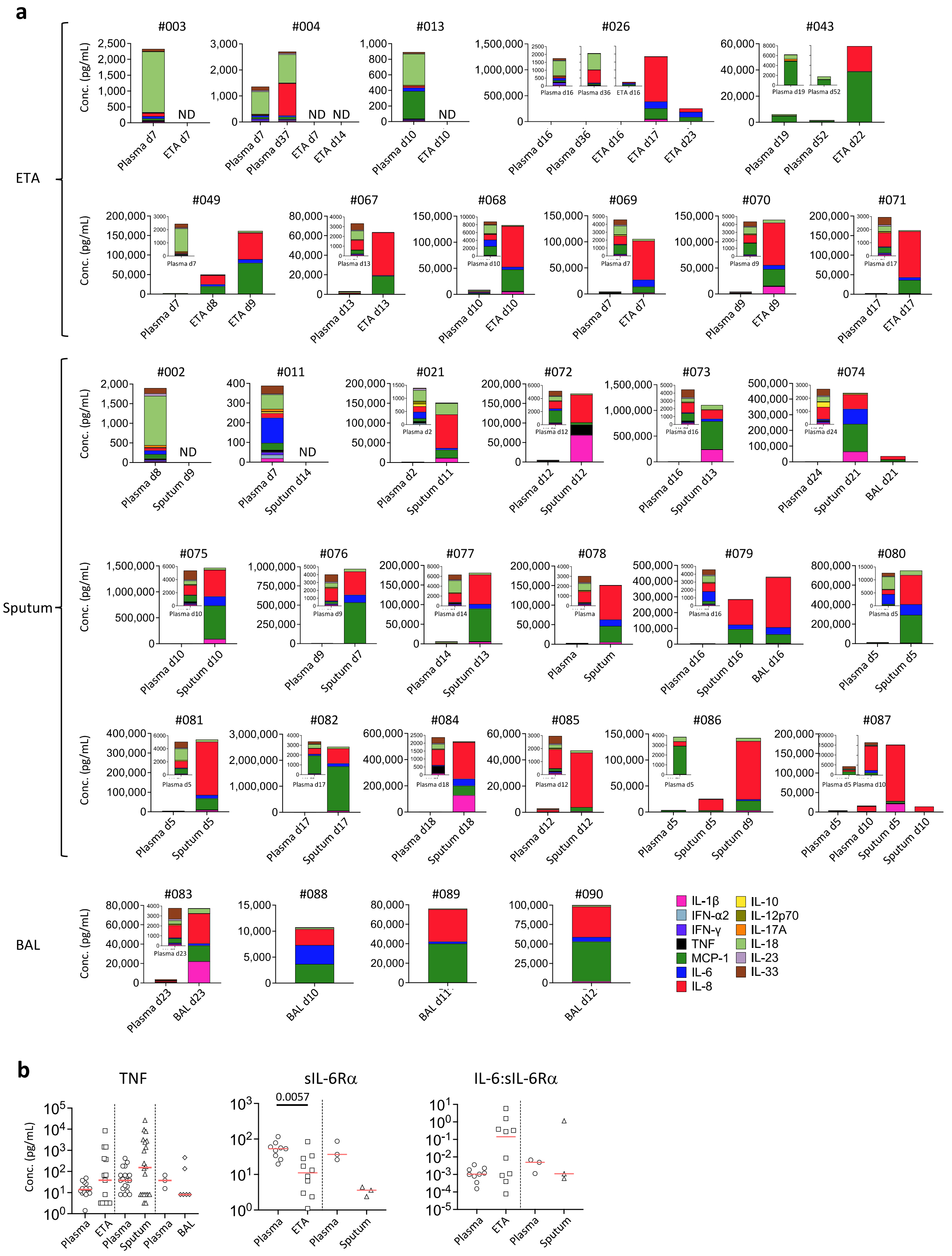
a
ETA
Sputum
BAL
Conc. (pg/mL)
#003
#004
#013
#026
#043
#049
#067
#068
#069
#070
#071
#002
#011
#021
#072
#073
#074
#075
#076
#077
#078
#079
#080
#081
#082
#084
#085
#086
#087
#083
#088
#089
#090
ND
IL-1β
IFN-α2
IFN-γ
TNF
MCP-1
IL-6
IL-8
IL-10
IL-12p70
IL-17A
IL-18
IL-23
IL-33
b
TNF
sIL-6Rα
IL-6:sIL-6Rα
0.0057
Plasma
ETA
Sputum
BAL

Supplementary Fig. 1 MCP-1, IL-6 and IL-8 dominate in COVID-19 respiratory samples. **(a)** Distribution of 13 cytokines and chemokines (IL-1β, IFN-α2, IFN-γ, TNF, MCP-1, IL-6, IL-8, IL-10, IL-12p70, IL-17A, IL-18, IL-23, and IL-33) in each COVID-19 patient that respiratory samples were collected. **(b)** Comparison of levels of TNF, soluble IL-6 receptor α (sIL-6Rα), and IL-6:sIL-6Rα ratio between plasma and respiratory samples for COVID-19 and non-COVID-19 patients. n_{ETA}=15, $n_{ETA\ matched\ plasma}$=14, n_{Sputum}=20, $n_{Sputum\ matched\ plasma}$=19, n_{BAL}=6, $n_{BAL\ matched\ plasma}$=3. Bars indicate median values. Statistical significance was determined with two-sided Mann-Whitney test. Source data are provided as a Source Data file.

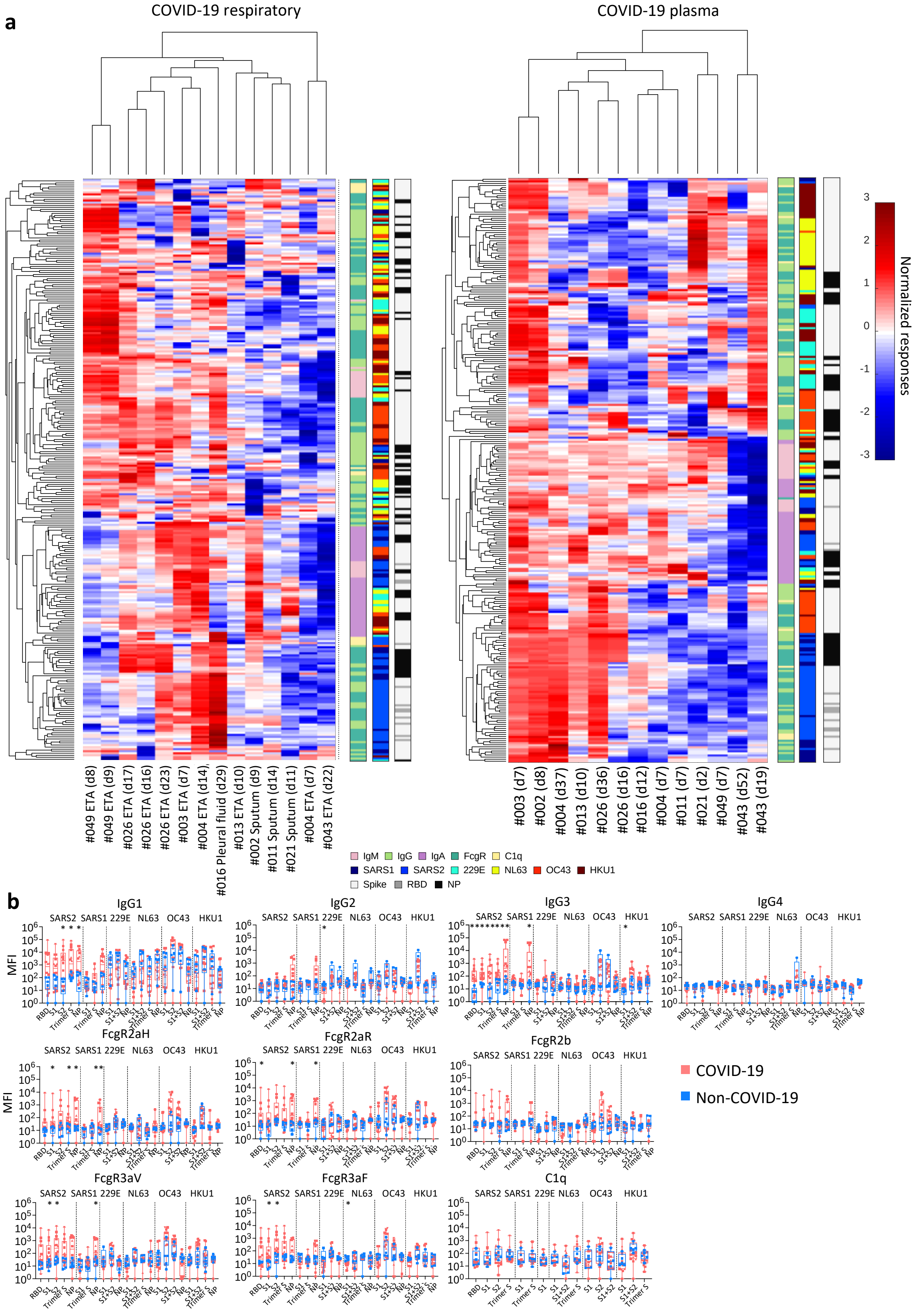
a
COVID-19 respiratory
COVID-19 plasma
Normalized responses
#049 ETA (d8)
#049 ETA (d9)
#026 ETA (d17)
#026 ETA (d16)
#026 ETA (d23)
#003 ETA (d7)
#004 ETA (d14)
#016 Pleural fluid (d29)
#013 ETA (d10)
#002 Sputum (d9)
#011 Sputum (d14)
#021 Sputum (d11)
#004 ETA (d7)
#043 ETA (d22)
#003 (d7)
#002 (d8)
#004 (d37)
#013 (d10)
#026 (d36)
#026 (d16)
#016 (d12)
#004 (d7)
#011 (d7)
#021 (d2)
#049 (d7)
#043 (d52)
#043 (d19)
IgM IgG IgA FcgR C1q
SARS1 SARS2 229E NL63 OC43 HKU1
Spike RBD NP
b
IgG1
IgG2
IgG3
IgG4
FcgR2aH
FcgR2aR
FcgR2b
FcgR3aV
FcgR3aF
C1q
SARS2 SARS1 229E NL63 OC43 HKU1
MFI
COVID-19
Non-COVID-19

Supplementary Fig. 2 Higher SARS-CoV-2-specific antibodies in COVID-19 than non-COVID-19 respiratory samples. (a) Heatmaps with unsupervised clustering of antibodies against receptor binding domain (RBD), spike proteins (S), and nucleoprotein (NP) of SARS-CoV-2 (SARS2) and other human coronaviruses including SARS-CoV-1 (SARS1), 229E, NL63, OC43, and HKU1 in COVID-19 plasma and respiratory samples (endotracheal tube aspirate (ETA), sputum, or pleural fluid), as measured by multiplex bead array assay. **(b)** Median fluorescence intensity of IgG1-4 and antibodies with FcγR2aH, FcγR2aR, FcγR2b, FcγR3aV, FcγR3aF, or C1q binding abilities between COVID-19 and non-COVID-19 respiratory samples. The bounds of the box plot indicate the 25th and 75th percentiles, the bar indicates medians, and the whiskers indicate minima and maxima. Statistical significance was determined with two-sided Mann-Whitney test. The P values for IgG1 against SARS2 S2, SARS2 Trimer S and SARS2 NP are 0.0352, 0.0194, 0.0194 respectively. The P value for IgG2 against 229E S1 is 0.0314. The P values for IgG3 against SARS2 RBD, SARS2 S1, SARS2 S2, SARS2 Trimer S, SARS2 NP, SARS1 NP and HKU1 S1 are 0.0070, 0.0437, 0.0052, 0.0064, 0.0103, 0.0419, 0.0357 respectively. The P values for antibodies with FcgR2aH binding ability against SARS2 S1, SARS2 Trimer S, SARS2 NP and SARS1 NP are 0.0454, 0.0339, 0.0455, 0.0076 respectively. The P values for antibodies with FcgR2aR binding ability against SARS2 RBD, SARS2 NP and SARS1 NP are 0.0461, 0.0194, 0.0437 respectively. The P values for antibodies with FcgR3aV binding ability against SARS2 S1, SARS2 S2 and SARS1 NP are 0.0437, 0.0437, 0.0339 respectively. The P values for antibodies with FcgR3aF binding ability against SARS2 S1, SARS2 S2 and NL63 S1 are 0.0437, 0.0324, 0.0147 respectively. $n_{\text{COVID-19 ETA}}=10$, $n_{\text{COVID-19 Sputum}}=3$, $n_{\text{COVID-19 pleural fluid}}=1$, $n_{\text{Respiratory matched COVID-19 plasma}}=13$, $n_{\text{Non-COVID-19 ETA}}=5$, $n_{\text{Non-COVID-19 sputum}}=1$. Source data are provided as a Source Data file.

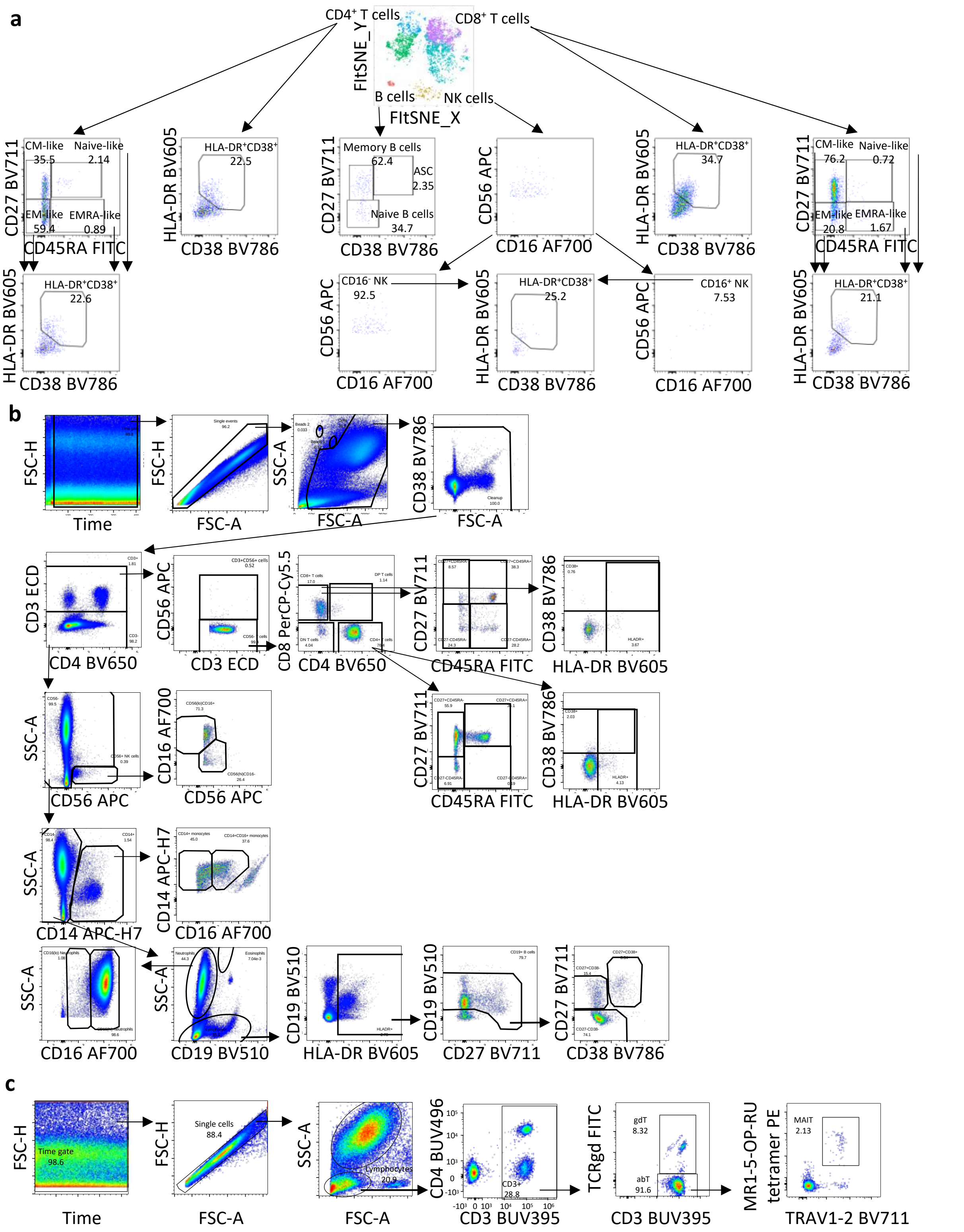

Supplementary Fig. 3 Gating strategies for flow cytometry analyses. (a) Respiratory myeloid antibody panel. **(b)** Whole blood lymphocyte antibody panel. **c** Whole blood innate T cell panel.

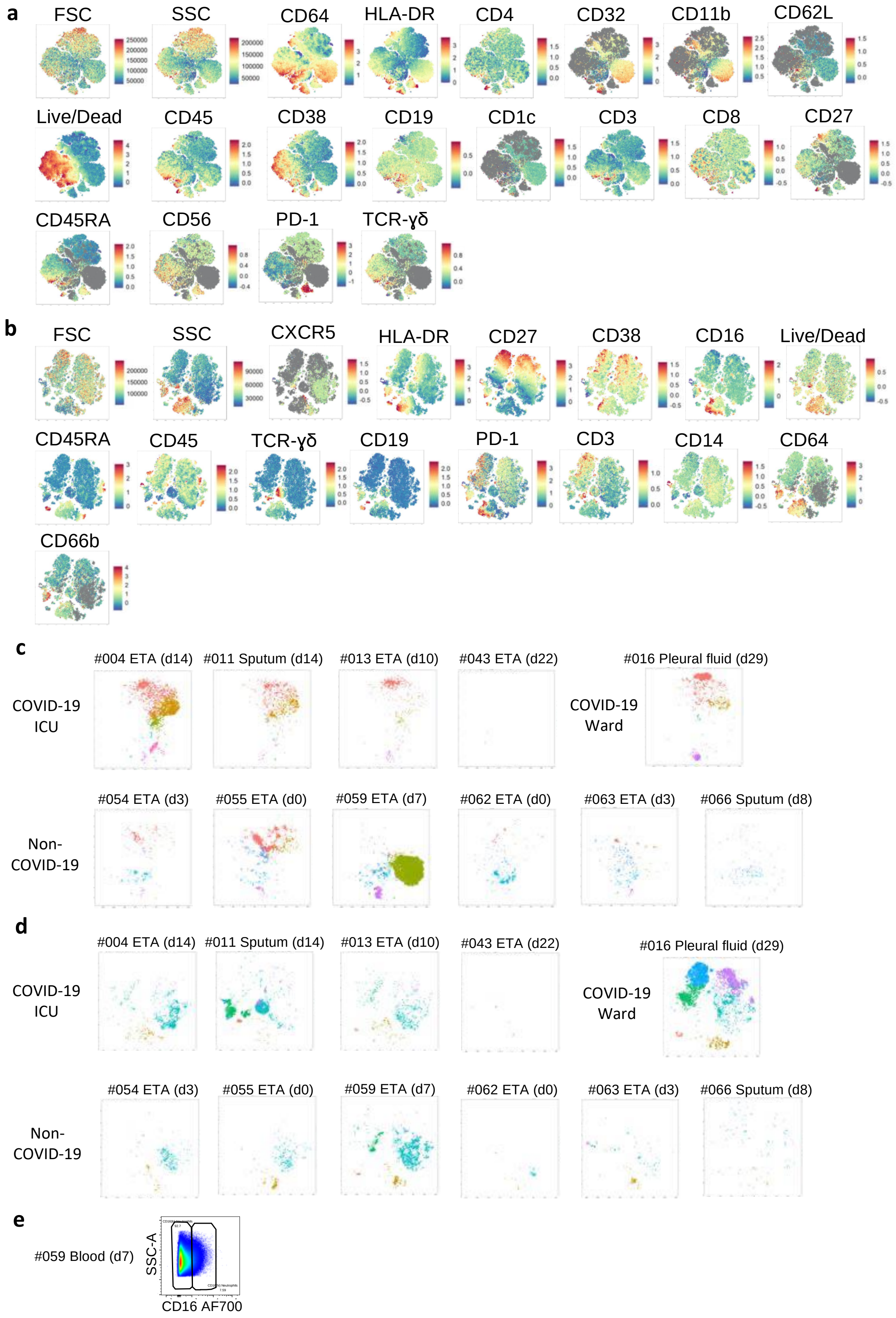
a
FSC
SSC
CD64
HLA-DR
CD4
CD32
CD11b
CD62L
Live/Dead
CD45
CD38
CD19
CD1c
CD3
CD8
CD27
CD45RA
CD56
PD-1
TCR-γδ
b
FSC
SSC
CXCR5
HLA-DR
CD27
CD38
CD16
Live/Dead
CD45RA
CD45
TCR-γδ
CD19
PD-1
CD3
CD14
CD64
CD66b
c
COVID-19 ICU
#004 ETA (d14)
#011 Sputum (d14)
#013 ETA (d10)
#043 ETA (d22)
COVID-19 Ward
#016 Pleural fluid (d29)
Non-COVID-19
#054 ETA (d3)
#055 ETA (d0)
#059 ETA (d7)
#062 ETA (d0)
#063 ETA (d3)
#066 Sputum (d8)
d
COVID-19 ICU
#004 ETA (d14)
#011 Sputum (d14)
#013 ETA (d10)
#043 ETA (d22)
COVID-19 Ward
#016 Pleural fluid (d29)
Non-COVID-19
#054 ETA (d3)
#055 ETA (d0)
#059 ETA (d7)
#062 ETA (d0)
#063 ETA (d3)
#066 Sputum (d8)
e
#059 Blood (d7)
SSC-A
CD16 AF700

Supplementary Fig. 4 Flow Self-Organizing Map (FlowSOM) analyses of respiratory samples. (a-b) Cell surface marker expression of **(a)** respiratory myeloid antibody panel and **(b)** respiratory lymphocyte antibody panel. **(c-d)** Individual Fit-SNE plots of the **c** myeloid antibody panel and **d** lymphocyte antibody panel. (**e)** Representative flow cytometry plot of #059 blood neutrophils.

Supplementary Table 1 COVID-19 cohort demographics

Patient	Age	Sex	Days post disease onset#			Days in hospital	Location during hospitalization	Oxygen supply	Drug therapy
			V1	HS	V7				
001	71-80	M		19		30	Ward	NP/HM	D+R
002R	41-50	M		8		9	ICU	NP	D+R
003R	41-50	M	7			18	ICU	ETT	D+R
004R	61-70	F	7		37	31	ICU	ETT	D
005	71-80	F	3			6	Ward	NP	D+R
006	31-40	F	11		12	2	Ward	N	N
007	21-30	F	7		8	2	Ward	N	N
008	21-30	F	11		12	2	Ward	N	N
009	51-60	F	7		8	2	Ward	N	N
010	51-60	M	11			6	Ward	N	D
011R	41-50	F	7			27	ICU	NP	D+R
012	61-70	M	9			13	ICU	NP	D+R
013R	61-70	M	10			12	ICU	ETT	D+R
014	31-40	M	10			6	Ward	N	D
015	21-30	M	5			2	Ward	N	N
016	61-70	M	12			9	Ward	N	N
017	71-80	M	13			4	Ward	N	N
018	71-80	F	7			4	Ward	N	N
019	21-30	M	5		16	11	Ward	N	D+R
020	71-80	M	5			9	Ward	NP	D+R
021R	61-70	F	2			19	Ward	NP/HM	D
022	51-60	M	7		10	4	Ward	N	N
023	51-60	M	8		15	9	ICU	NP/HM	D+R
024	61-70	F	12			4	Ward	N	N
025	51-60	F	5		26	21	ICU	HFNP	D
026R	51-60	F	16		36	29	ICU	ETT	D
027	51-60	F	6			3	Ward	N	N
028	31-40	F	12		17	6	Ward	N	N
029	61-70	M	7		28	23	Ward	HFNP	D
030	71-80	F	12		15/18##	47	ICU	N	N
031	81-90	M	2		10	24	Ward	N	N
032	31-40	M	11			1	Ward	N	N
033	21-30	F	9			1	Ward	N	N
034	41-50	F	8		11	4	Ward	NP	D+R
035	21-30	F	13			2	Ward	N	D
036	61-70	M	9			2	Ward	N	N
037	51-60	F	9			7	Ward	N	D
038	61-70	F	9		12	4	Ward	NP	D
039	81-90	M	9		14	5	Ward	NP	D+R
040	71-80	M	4		27	25	Ward	NP	D
041	31-40	M	6			1	Ward	N	N
042	21-30	M	7		12	4	Ward	NP	D+R
043R	61-70	M		19	52	44	ICU	ETT	D
044	71-80	F	3		10/16##	17	ICU	NP	D+R
045	51-60	M	7			4	ICU	NP	D+R
046	81-90	M	15		24	11	Ward	N	N
047	71-80	M	8			6	Ward	NP	D
048	51-60	M	10		17	7	ICU	NP	D
049R	21-30	F	7			6	ICU	ETT	D+R
050	51-60	F	7			12	Ward	NP	D+R
051	71-80	F	14		17	5	Ward	NP	N
052	51-60	F	11		16	6	Ward	N	N
053	81-90	M	6		15	13	Ward	N	D
054R*	41-50	F	3^		53	51	ICU	ETT	N
055R*	71-80	F	0^		19	19	ICU	ETT	N
056	51-60	M	35		41	8	ICU	NP	D
057	31-40	F	10		11	2	Ward	N	N

058	51-60	M	12		20	10	Ward	NP	D
059[R]*	81-90	M	7			22	ICU	ETT	N
060	41-50	F	13		16	4	Ward	N	N
061	71-80	M	0			7	Ward	NP	D
062[R]*	61-70	M	0^			13	ICU	ETT	N
063[R]*	81-90	M	3			10	ICU	ETT	N
064	41-50	M				7	ICU	HFNP	D+R
065	41-50	F	5			1	Ward	N	N
066[R]*	61-70	F	8			3	Ward	NP	N
067[R]	31-40	F		13		21	ICU	ETT	D+R
068[R]	41-50	M		10		27	ICU	ETT	D
069[R]	61-70	M		7		24	ICU	ETT	D
070[R]	31-40	M		9		23	ICU	ETT	D
071[R]	51-60	M		17		17	ICU	ETT	D+R
072[R]	21-30			12			ICU	ETT	D
073[R]	61-70			16			ICU	ETT	D
074[R]	51-60			24			ICU	ETT	D+R
075[R]	51-60			10			ICU	ETT	D
076[R]	61-70			9			ICU	HFNP	D+R
077[R]	51-60			14			ICU	ETT	D
078[R]	51-60						ICU	ETT	D+R
079[R]	21-30			16			ICU	ETT	R
080[R]	71-80			5			ICU	ETT	D
081[R]	71-80			5			ICU	ETT	D
082[R]	51-60			17			ICU	ETT	D+R
083[R]	41-50			23			ICU	ETT	D+R
084[R]	61-70			18			ICU	ETT	D
085[R]	41-50	F		12		4	ICU	ETT	D
086[R]	61-70	M		5		5	Ward	N	
087[R]	51-60	M		5/10		4	Ward	N	
088[R]	61-70	M				24	ICU	ETT	N
089[R]	31-40	F				26	ICU	ETT	N
090[R]	61-70	M				43	ICU	ETT	D

[#]Days post disease onset of blood samples collection. [R]Patients with respiratory samples collected. *Non-COVID-19 patients with variable disease: 054, decompensated alcoholic hepatitis with encephalopathy; 055, hemangioblastoma intracranial haemorrhage; 059, Klebsiella pneumonia; 062, intracranial haemorrhage; 063, atypical pneumonia; 066, infective exacerbation of chronic obstructive pulmonary disease. ^Date of intubation was used because of no respiratory disease onset. [##]Patients with delays after anticipated discharge date, data from the later sample were used for analyses if available. V1, hospital admission; HS, hospital stay; V7, hospital discharge. In patients 072-084, 3 were female. Abbreviations: N, none; NP, nasal prong; NP/HM, nasal prong/ Hudson mask; HFNP, high flow nasal prong; ETT, endotracheal tube; D, dexamethasone; R, remdesivir; D+R, dexamethasone and remdesivir.

Supplementary Table 2 Clinical summary of COVID-19 cohort

COVID-19 positive patients	Total (n=84)	Ward (n=45)	ICU (n=39)	p value[a]
**Age** (years), median (range)	56.5 (22-90)	58 (22-90)	56 (25-78)	0.5814[c]
**Female**, n (%)	31 (36.9%)	21 (46.7%)	10 (25.6%)	0.2673[d]
**Ethnicity**, n (%)				0.7936[d, e]
African	3 (3.6%)	1 (2.2%)	2 (5.1%)	
Arabic	3 (3.6%)	3 (6.7%)	0	
Asian	5 (6%)	4 (8.9%)	1 (2.6%)	
Aboriginal and Torres Strait Islander	1 (1.2%)	1 (2.2%)	0	
European	33 (39.3%)	24 (53.3%)	11 (28.2%)	
Indo-Asian	2 (2.4%)	2 (4.4%)	0	
Middle Eastern	7 (8.3%)	5 (11.1%)	3 (7.7%)	
Pacific Islander	1 (1.2%)	0	1 (2.6%)	
Samoan	1 (1.2%)	0	1 (2.6%)	
South Asian	4 (4.8%)	2 (4.4%)	2 (5.1%)	
Turkish	1 (1.2%)	1 (2.2%)	0	
Maori	1 (1.2%)	0	1 (2.6%)	
Unknown	19 (22.6%)	2 (4.4%)	17 (43.6%)	
**Weight** (kg), median (range)	78 (44.4-128.7)	75.5 (44.6-110)	91.8 (44.4-128.7)	0.0008[c]
**Height** (cm), median (range)	167 (152-193)	165 (152-193)	170 (157-185)	0.0476[c]
**BMI** (kg/m^2), median (range)	27.4 (17.4-43)	26.4 (17.4-41.4)	30.6 (18-43)	0.0085[c]
**Days from disease onset to hospitalization**, median (range)	7 (-1-48)	7 (-1-14)	6 (0-48)	0.4447[c]
**Days in hospital**, median (range)	7 (1-47)	5 (1-30)	19.5 (4-47)	<0.0001[c]
**Ward/ICU**, n (%)				N/A
Ward	45 (53.6%)	N/A	N/A	
ICU	39 (28.3%)	N/A	N/A	
**NIH score**, n (%)				<0.0001[d, f]
2	6 (7.14%)	6 (13.3%)	0	
3	21 (25%)	20 (44.4%)	1 (2.6%)	
4	27 (32.1%)	16 (35.6%)	11 (28.2%)	
5	11 (13.1%)	1 (2.2%)	10 (25.6%)	
**Oxygen support**, n (%)				<0.0001[d, g]
**None**	31 (36.9%)	30 (66.7%)	1 (2.6%)	
**Non-Invasive**	26 (31%)	15 (33.3%)	11 (28.2%)	
Nasal prong	19 (22.6%)	12 (26.7%)	7 (17.9%)	
Nasal prong / Hudson mask	3 (3.6%)	2 (4.4%)	1 (2.6%)	
High flow nasal prong	4 (4.8%)	1 (2.2%)	3 (7.7%)	
**Invasive**	27 (32.1%)	0	27 (69.2%)	
Endotracheal tube	27 (32.1%)	0	27 (69.2%)	
**Clinical presentation** (ILI/pneumonia/chest x-ray consolidation), n (%)				0.3142[d]
None	13 (15.5%)	11 (24.4%)	2 (5.1%)	
Yes	47 (56%)	32 (71.1%)	15 (38.5%)	
Unknown	24 (28.6%)	2 (4.4%)	22 (56.4%)	
**Drugs**, n (%)				<0.0001[d, g]
None	26 (31%)	23 (51.1%)	3 (7.7%)	
Dexamethasone (5-day course)	30 (35.7%)	12 (26.7%)	18 (46.2%)	
Dexamethasone (5-day course) + Remdesivir (5-day course)	25 (30%)	8 (17.8%)	17 (43.6%)	
Remdesivir	1 (1.2%)	0	1 (2.6%)	
Unknown	2 (2.4%)	2 (4.4%)	0	
**Immunosuppressants**, n (%)				0.7063[d, g]
None	57 (67.9%)	37 (82.2%)	20 (51.3%)	
Ciclosporin + Mycophenolate mofetil	1 (1.2%)	1 (2.2%)	0	
Vinblastine + Prednisolone + Pembrolizumab	1 (1.2%)	1 (2.2%)	0	
Methotrexate + Prednisolone	1 (1.2%)	0	1 (2.6%)	
Prednisolone	3 (3.6%)	2 (4.4%)	1 (2.6%)	
Dexamethasone	1 (1.2%)	1 (2.2%)	0	
Tacrolimus	1 (1.2%)	1 (2.2%)	0	
Tacrolimus + Mycophenolate mofetil	0	0	0	
Unknown	19 (22.6%)	2 (4.4%)	17 (43.6%)	
**Smoker**, n (%)				0.1844[d, h]
Non-smoker	44 (52.4%)	31 (68.9%)	13 (33.3%)	
Ex-smoker	8 (9.5%)	7 (15.6%)	1 (2.6%)	
Smoker	7 (8.3%)	3 (6.7%)	4 (10.3%)	
Unknown	25 (29.8%)	4 (8.9%)	21 (53.8%)	

[a]Comparison between COVID-19 positive ward and ICU patients.
[b]Comparison between COVID-19 positive and COVID-19 negative patients.
[c]Significance was determined using the two-sided Mann-Whitney test.
[d]Significance was determined using the two-sided Fisher's exact test.
[e]Comparison between European and other ethnicities.

[f]Comparison between NIH score 2, 3 and NIH score 4, 5.
[g]Comparison between patients with or without oxygen support, drug treatments, or immunosuppressants.
[h]Comparison between Non-smoker and Ex-smoker, and Smoker.
Weight, height, BMI, days from disease onset to hospitalization and NIH score unknown for 19 patients. Days in hospital unknown for 13 patients. Unknown datapoints were not included in statistical analyses.
Abbreviations: ICU, intensive care unit; N/A, not applicable; BMI, body mass index; NIH, National Institutes of Health; ILI, influenza-like-illness.

Supplementary Table 3 Grading criteria of the National Institutes of Health (NIH) score*

NIH score	Criteria
Asymptomatic/ Presymptomatic (1)	Individuals who:<br>• test positive for SARS-CoV-2 by virologic testing, and<br>• have no symptoms
Mild (2)	Individuals who:<br>• have COVID-19 symptoms such as fever, cough, sore throat, malaise, headache, muscle pain, and<br>• without shortness of breath, dyspnoea, or abnormal chest imaging
Moderate (3)	Individuals who:<br>• have evidence of lower respiratory disease by clinical assessment or imaging, and<br>• have a saturation of oxygen (SpO_2) ⩾94% on room air at sea level
Severe (4)	Individuals who:<br>• have respiratory frequency >30 breaths per minute, or<br>• SpO_2 <94% on room air at sea level, or<br>• ratio of arterial partial pressure of oxygen to fraction of inspired oxygen (PaO_2/FiO_2) <300 mmHg, or<br>• lung infiltrates >50%
Critical (5)	Individuals who:<br>• have respiratory failure, septic shock, and/or multiple organ dysfunction

*https://www.covid19treatmentguidelines.nih.gov/overview/clinical-spectrum/

Supplementary Table 4 Validation test of the surrogate Virus Neutralization Test

Sample	COVID-19 status	Dilution[a]	% Inhibition[b]
COVID-19 positive serum	Positive	Neat	95.6
		1:2	86.4
		1:10	44.1
055 ETA	Negative	Neat	10.5
		1:2	87.3
		1:10	43.1
066 Sputum	Negative	Neat	0
		1:2	86.9
		1:10	40.1

[a]COVID-19 negative ETA and sputum were tested in neat or mixed with the COVID-19 positive serum until the COVID-19 positive serum was in the dilution as stated in the column.
[b]Positive % inhibition was defined as ⩾ 20%.
Abbreviation: ETA, endotracheal aspirate.

Supplementary Table 5 Panel design of the multiplex bead array assay

Pathogens	Proteins	Isotypes and FcɣR/C1q bindings
SARS-CoV-2	RBD	IgG
	S1	IgG1
	S2	IgG2
	Trimeric S	IgG3
	NP	IgG4
SARS-CoV-1	S1	IgA1
	Trimeric S	IgA2
	NP	IgM
HCoV 229E	S1	FcɣR2aH
	S1+2	FcɣR2aR
	NP	FcɣR2b
HCoV NL63	S1	FcɣR3aV
	S1+2	FcɣR3aF
	Trimeric S	C1q[a]
	NP	
HCoV OC43	S1	
	S2	
	S1+2	
	NP	
HCoV HKU1	S1	
	S1+2	
	Trimeric S	
	NP	
C. Tetani	Tetanus Toxin	
Influenza A/Cali/07/2009 (H1N1)	Hemagglutinin	

[a]C1q binding was not tested for NP of SARS-CoV-2, SARS-CoV-1, HCoV 229E, HCoV NL63, HCoV OC43 and HCoV HKU1, and HCoV 229E S1+2. Abbreviations: FcɣR, fragment crystallizable region gamma receptor; C1q, complement component 1q; RBD, receptor binding domain; S, spike; NP, nucleoprotein; HCoV, human coronavirus.

Supplementary Table 6 Detectable sIL-6Ra, ADAMTS4 and antibody levels in 6 respiratory samples with undetectable cytokine/chemokine levels

Sample	sIL6Ra	ADAMTS4	IgM	IgG	IgA	% inhibition sVNT
002 Sputum	2.381	15.625	2.645	1.239	4.101	3.448
003 ETA	2.970	1264.532	3.445	3.686	3.618	28.966
004 ETA d7	2.360	15.625	0.000	1.646	1.772	3.448
004 ETA d14	33.553	15.625	3.796	4.321	4.081	48.828
011 Sputum	4.404	15.625	2.456	2.818	2.366	0.690
013 ETA	6.613	15.625	1.296	2.209	2.622	0.000

Please refer to the “Source data” file for the complete database.

Supplementary Table 7 Flow cytometry antibody panels

Colour	Fluorochrome	Respiratory myeloid panel	Respiratory lymphocyte panel	Whole blood lymphocyte panel	Whole blood innate T cell panel
Violet	BV421	CD66b	CXCR5	CD71	
	BV510	CD64		CD19	Live/Dead
	BV605	HLA-DR	HLA-DR	HLA-DR	CD161
	BV650	CD4	CD4	CD4	
	BV711	CD32	CD27	CD27	TRAV1-2
	BV786	CD11b	CD38	CD38	
Red	APC	CD62L	CD56	CD56	
	AF700	CD16	CD16	CD16	CD27
	APC-H7	Live/Dead	Live/Dead	CD14	CD19
Blue	FITC		CD45RA	CD45RA	TCRgd
	PerCP-Cy5.5	CD45	CD45	CD8	
Yellow-Green	PE	CD38	TCRgd	TCRgd	MR1-5-OP-RU tetramer
	ECD	CD19	CD19	CD3	
	PE-Cy7	CD1c	PD-1		
UV	BUV395	CD3	CD3		CD3
	BUV496				CD4
	BUV737	CD14	CD14		
	BUV805	CD8	CD8		CD8
