# Supplementary material for: SARS-CoV-2 infection results in immune responses in the respiratory tract and peripheral blood that suggest mechanisms of disease severity
Source: Nat Commun. 2022 May 19;13:2774. doi: 10.1038/s41467-022-30088-y (PMC9120039; doi:10.1038/s41467-022-30088-y)
Supplement: Supplementary file 3 — Reporting Summary [file 41467_2022_30088_MOESM3_ESM.pdf]

nature portfolio

Corresponding author(s): Katherine Kedzierska, Oanh Nguyen, Claire Gordon, Jason Trubiano

Last updated by author(s): 2022/03/08

Reporting Summary

Nature Portfolio wishes to improve the reproducibility of the work that we publish. This form provides structure for consistency and transparency in reporting. For further information on Nature Portfolio policies, see our Editorial Policies and the Editorial Policy Checklist.

Statistics

For all statistical analyses, confirm that the following items are present in the figure legend, table legend, main text, or Methods section.

n/a	Confirmed	
☐	☒	The exact sample size (*n*) for each experimental group/condition, given as a discrete number and unit of measurement
☐	☒	A statement on whether measurements were taken from distinct samples or whether the same sample was measured repeatedly
☐	☒	The statistical test(s) used AND whether they are one- or two-sided<br>*Only common tests should be described solely by name; describe more complex techniques in the Methods section.*
☐	☒	A description of all covariates tested
☐	☒	A description of any assumptions or corrections, such as tests of normality and adjustment for multiple comparisons
☐	☒	A full description of the statistical parameters including central tendency (e.g. means) or other basic estimates (e.g. regression coefficient) AND variation (e.g. standard deviation) or associated estimates of uncertainty (e.g. confidence intervals)
☐	☒	For null hypothesis testing, the test statistic (e.g. *F*, *t*, *r*) with confidence intervals, effect sizes, degrees of freedom and *P* value noted<br>*Give P values as exact values whenever suitable.*
☒	☐	For Bayesian analysis, information on the choice of priors and Markov chain Monte Carlo settings
☒	☐	For hierarchical and complex designs, identification of the appropriate level for tests and full reporting of outcomes
☒	☐	Estimates of effect sizes (e.g. Cohen's *d*, Pearson's *r*), indicating how they were calculated

Our web collection on statistics for biologists contains articles on many of the points above.

Software and code

Policy information about availability of computer code

Data collection	BD FACS DIVA v8.0.1; Thermo Ascent Software for Multiskan v2.4; Luminex xPONENT v4.3
Data analysis	FlowJo v10; Prism v8, v9; R v3.5.3, v3.6.2, v4.0.2; IQ-Tree v2.1.-, options "-mset GTR+G4 -bb 1000"; ggtree package (v.1.14.6); Spectre v0.4.1; FlowSOM v1.20.0; Fit-SNE v1.2.1; Matlab 2017b; Eigenvectors PLS toolbox 8.2; LEGENDplex™ Data Analysis Software v7.1

For manuscripts utilizing custom algorithms or software that are central to the research but not yet described in published literature, software must be made available to editors and reviewers. We strongly encourage code deposition in a community repository (e.g. GitHub). See the Nature Portfolio guidelines for submitting code & software for further information.

Data

Policy information about availability of data

All manuscripts must include a data availability statement. This statement should provide the following information, where applicable:

- Accession codes, unique identifiers, or web links for publicly available datasets
- A description of any restrictions on data availability
- For clinical datasets or third party data, please ensure that the statement adheres to our policy

All data generated or analysed during this study are included in this published article (and its supplementary information files). Source data are provided with this paper as Source Date file 'Source data file_Drastic.xlsx'. All relevant data are also available from the authors. The viral sequences isolated from nasal swabs that support the findings of this study are available on the GISAID database with ID numbers provided in the Source data file. Registration to access the database is free and open to anyone using the link: https://www.gisaid.org/registration/register/.

Field-specific reporting

Please select the one below that is the best fit for your research. If you are not sure, read the appropriate sections before making your selection.

- [x] Life sciences
- [ ] Behavioural & social sciences
- [ ] Ecological, evolutionary & environmental sciences

For a reference copy of the document with all sections, see nature.com/documents/nr-reporting-summary-flat.pdf

Life sciences study design

All studies must disclose on these points even when the disclosure is negative.

Sample size	The sample size of endotrachial aspirate and bronchoalveolar lavage was determined by the availability of samples from intubated COVID-19 patients in Victoria and New South Wales, Australia in 2020-2021. The sample size of blood was determined by the availability of samples from hospitalized COVID-19 patients in Victoria and New South Wales, Australia in 2020-2021. The sample size of Non-COVID-19 endotrachial aspirate was determined by the availability of samples from intubated patients in Victoria, Australia in 2020-2021 with negative SARS-CoV-2 PCR results. The sample size of Non-COVID-19 sputum and blood was determined by the availability of samples from hospitalized patients in Victoria, Australia in 2020-2021 with negative SARS-CoV-2 PCR results.
Data exclusions	No data were excluded. A source data file is provided to show all data points.
Replication	Experiments could not be replicated due to limited frozen cells from respiratory samples. These are rare and precious patient samples and so we were limited to performing all the available assays. To ensure reliability, all timepoints from the same patient were carried out in the same experiment.
Randomization	Randomization was not applicable to the study. The study analyzed data of available samples from hospitalized patients receiving a positive or negative SARS-CoV-2 PCR result. Other covariates were not controlled in the study as the samples are rare and precious and we did not exclude anyone willing to participate in the study.
Blinding	Experiments were not blinded.

Reporting for specific materials, systems and methods

We require information from authors about some types of materials, experimental systems and methods used in many studies. Here, indicate whether each material, system or method listed is relevant to your study. If you are not sure if a list item applies to your research, read the appropriate section before selecting a response.

Materials & experimental systems

n/a	Involved in the study	
☐	☒	Antibodies
☐	☒	Eukaryotic cell lines
☒	☐	Palaeontology and archaeology
☒	☐	Animals and other organisms
☐	☒	Human research participants
☒	☐	Clinical data
☒	☐	Dual use research of concern

Methods

n/a	Involved in the study	
☒	☐	ChIP-seq
☐	☒	Flow cytometry
☒	☐	MRI-based neuroimaging

Antibodies

Antibodies used

We used commercially-available antibodies as per Material and Methods, and Supplementary Table 5, 7.
SARS-CoV-2 RBD ELISA were performed with Human IgM-Biotin (Mabtech, #3880-6-1000, MT22, 1:1000), Human IgG Fcg-HRP (Jackson ImmunoResearch, #109-035-008, polyclonal, 1:10000), Human IgA-ALP (Mabtech, #3860-9A-1000, MT20, 1:1000).
Antibodies for cytokine analysis were within commercial kit (BioLegend, #740809).
Antibodies for sIL-6a (R&D Systems, #DY227) and ADAMTS4 (R&D Systems, #DY4307-05) ELISAs were within commercial kits.
Luminex bead-based multiplex assay used the following detection antibodies: Human IgG Fc-PE (Southern Biotech, #9040-09, clone JDC-10, 1.3µg/ml), Human IgG1 Hinge-PE (Southern Biotech, #9052-09, clone 4E3, 1.3µg/ml), Human IgG2 Fc-PE (Southern Biotech, #9070-09, clone HP6002, 1.3µg/ml), Human IgG3 Hinge-PE (Southern Biotech, #9210-09, clone HP6050, 1.3µg/ml), Human IgG4 Fc-PE (Southern Biotech, #9200-09, clone HP6025, 1.3µg/ml), Human IgA1-PE (Southern Biotech, #9130-09, clone B3506B4, 1.3µg/ml), Human IgA2- PE (Southern Biotech, #9140-09, clone A9604D2, 1.3µg/ml), Human IgM-Biotin (Mabtech, #3880-6-250, MT22, 1.3µg/ml).
Respiratory myeloid panel was stained with BV421 CD66b (BD Horizon, #562940, clone G10F5, 1:100), BV510 CD64 (BD Horizon, #563459, clone 10.1, 1:100), BV605 HLA-DR (Biolegend, #307640, clone L243, 1:50), BV650 CD4 (BD Biosciences, #563875, clone SK3, 1:200), BV711 CD32 (BD Horizon, #564839, FLI8-26, 1:100), BV785 CD11b (BioLegend, #301346, clone ICRF44, 1:100), APC CD62L (BD Pharmingen, #561916, clone DREG-56, 1:50), AF700 CD16 (BioLegend, #302026, clone 3G8, 1:50), APC-H7 Live/Dead Near-IR (ThermoFisher Scientific, #L34976, 1:1000), PerCP-Cy5.5 CD45 (BD, #340953, clone 2D1, 1:50), PE CD38 (BD Pharmingen, #555460,

clone HIT2, 1:50), ECD CD19 (Beckman Coulter, #IM2708U, clone J3-119, 1:150), PE-Cy7 CD1c (BioLegend, #331516, clone L161, 1:200), BUV395 CD3 (BD Horizon, #563546, clone UCHT1, 1:200), BUV737 CD14 (BD Horizon, #564444, clone M5E2, 1:200), BUV805 CD8 (BD Horizon, #564912, clone SK1, 1:200).

Respiratory lymphocyte panel was stained with BV421 CXCR5 (BD Horizon, #562747, clone RF8B2, 1:50), BV605 HLA-DR (Biolegend, #307640, clone L243, 1:50), BV650 CD4 (BD Biosciences, #563875, clone SK3, 1:200), BV711 CD27 (BD Horizon, #563167, clone L128, 1:200), BV786 CD38 (BD Horizon, #563964, clone HIT2, 1:200), APC CD56 (BioLegend, #304610, clone MEM-188, 1:50), AF700 CD16 (BioLegend, #302026, clone 3G8, 1:50), APC-H7 Live/Dead Near-IR (ThermoFisher Scientific, #L34976, 1:1000), FITC CD45RA (BD Pharmingen, #555488, clone HI100, 1:50), PerCP-Cy5.5 CD45 (BD, #340953, clone 2D1, 1:50), PE TCRgd (BD, #347907, clone 11F2, 1:100), ECD CD19 (Beckman Coulter, #IM2708U, clone J3-119, 1:150), PE-Cy7 PD-1 (BD Pharmingen, #561272, clone EH12.1, 1:100), BUV395 CD3 (BD Horizon, #563546, clone UCHT1, 1:200), BUV737 CD14 (BD Horizon, #564444, clone M5E2, 1:200), BUV805 CD8a (BD Horizon, #564912, clone SK1, 1:200).

Whole blood lymphocyte panel was stained with BV421 CD71 (BD Horizon, #562995, clone M-A712, 1:50), BV510 CD19 (BD Horizon, #562947, clone SJ25C1, 1:200), BV605 HLA-DR (Biolegend, #307640, clone L243, 1:50), BV650 CD4 (BD Biosciences, #563875, clone SK3, 1:200), BV711 CD27 (BD Horizon, #563167, clone L128, 1:200), BV786 CD38 (BD Horizon, #563964, clone HIT2, 1:400), APC CD56 (BioLegend, #304610, clone MEM-188, 1:50), AF700 CD16 (BioLegend, #302026, clone 3G8, 1:50), APC-H7 CD14 (BD Pharmingen, #560180, clone MΦP9, 1:50), FITC CD45RA (BD Pharmingen, #555488, clone HI100, 1:50), PerCP-Cy5.5 CD8a (BD Biosciences, #565310, clone SK1, 1:200), PE TCRgd (BD, #347907, clone 11F2, 1:100), PE-CF594 CD3 (BD Biosciences, #562280, clone UCHT1, 1:800).

Whole blood innate T cell panel was stained with BV510 Live/Dead Aqua (Invitrogen, #L34957, 1:200), BV605 CD161 (Biolegend, #339916, clone HP-3G10, 1:100), BV711 TRAV1-2 (Biolegend, #560611, clone 3C10, 1:100), AF700 CD27 (BD Biosciences, #560611, clone M-T271, 1:50), APC-H7 CD19 (BD Biosciences, #557791, clone SJ25C1, 1:200), FITC TCRgd (BD Biosciences, #561995, clone B1, 1:50), PE MR1-5-OP-RU tetramer (refolded in-house, 1ug/ml, the validation of tetramer can be found in the following article https://doi.org/10.1038/nature13160), BUV395 CD3 (BD Biosciences, #563546, clone UCHT-1, 1:100), BUV496 CD4 (BD Biosciences, #612937, clone SK3, 1:200), BUV805 CD8 (BD Biosciences, #612889, clone SK1, 1:400).

Validation

Each antibody used had a validated technical data sheet as per manufacturer's website showing positive staining as opposed to the negative staining of isotype control. All antibodies were titrated in our laboratory prior to their use. ELISA and multiplex assay were tested with samples with known high responses from previous assays. Multiplex assay was also validated by testing single "beads" alone in parallel. FACS positive staining is shown for each antibody used as shown in the FACS plots in the main figures and supplementary figures.

Eukaryotic cell lines

Policy information about cell lines

Cell line source(s)	Vero cells were obtained from ATCC (#CCL-81).
Authentication	The cell line was not authenticated.
Mycoplasma contamination	Vero cells tested mycoplasma negative.
Commonly misidentified lines (See ICLAC register)	*Name any commonly misidentified cell lines used in the study and provide a rationale for their use.*

Human research participants

Policy information about studies involving human research participants

Population characteristics

Please refer to Supplementary Tables 1 (individual patient characteristics) and 2 (summarized patient characteristics) for details. In brief, for the 84 COVID-19 patients recruited in the study, 45 were hospitalized in ward and 39 required ICU admission. The patients had a median age of 56.5 with a range 22-90. 31 (36.9%) patients were female and the ethnicity of 33 (39.3%) patients were European. While 31 patients did not require oxygen support, 26 and 27 patients required non-invasive and invasive oxygen support respectively. 30, 25 and 1 patients received dexamethasone, dexamethasone and remdesivir, or remdesivir respectively.

Recruitment

Samples were recruited through the Austin Hospital, Royal Melbourne Hospital, Alfred Hospital in Victoria, Australia, and the Westmead Hospital in New South Wales, Australia. Patients admitted to the hospitals mentioned above were recruited to the study and SARS-CoV-2 PCR tests were performed. Signed informed consents were obtained from all donors prior to the study. There was no self-isolation bias or any other bias for recruiting donors to the study.

Ethics oversight

Experiments conformed to the Declaration of Helsinki Principles and the Australian National Health and Medical Research Council Code of Practice. Written informed consent was obtained from all donors prior to the study. The study was approved by the Austin Health (HREC/63201/Austin-2020), the Alfred Hospital Ethics Committee (Project 182/20), Western Sydney Local Area Health District (WSLHD) Human Research Ethics Committee (HREC) (2020/ETH00989), Melbourne Health (HREC/66341/MH-2020 and HREC/17/MH/53) and the University of Melbourne (#2057366.1, #2056901.1 and #1955465.3) Human Research Ethics Committees.

Note that full information on the approval of the study protocol must also be provided in the manuscript.

Flow Cytometry

Plots

Confirm that:

- [x] The axis labels state the marker and fluorochrome used (e.g. CD4-FITC).
- [x] The axis scales are clearly visible. Include numbers along axes only for bottom left plot of group (a 'group' is an analysis of identical markers).
- [x] All plots are contour plots with outliers or pseudocolor plots.
- [x] A numerical value for number of cells or percentage (with statistics) is provided.

Methodology

Sample preparation	Samples were prepared as described in Methods. For Figures 2-5, respiratory samples (ETA, sputum, BAL, or pleural fluid) were diluted in PBS and were filtered through a 45µm filter prior to separation of respiratory fluid and cellular contents by centrifugation. The cell pellet (ETA, sputum, or pleural fluid) was washed with EDTA-BSS and were stained with FcR block prior to staining for flow cytometry. For Figures 7-8, peripheral blood was collected in heparinized, ethylenediaminetetraacetic acid (EDTA) or serum tubes during hospitalization and centrifuged to collect plasma or serum. Whole blood was used for direct staining of immune cell subsets for flow cytometry.
Instrument	BD LSR Fortessa was used for acquisition of data
Software	BD FACS Diva v8.0.1, FlowJo v10.5.3
Cell population abundance	We have not sorted the samples.
Gating strategy	Gating strategy has been described in supplementary figure 3. For the respiratory myeloid panel, flow cytometry data was initially gated to exclude aberrantly acquired events, doublets, debris, and beads. The resulting cells were then exported for computational analysis. For the respiratory lymphocyte panel, flow cytometry data was initially gated to exclude aberrantly acquired events, doublets, debris, and beads. We then gated cells expressing combinations of CD3, CD4, CD8, CD19, CD56, and TCRgd. Cells expressing any combination of these markers was then exported for computational analysis. For the whole blood lymphocyte panel, flow cytometry data was initially gated to exclude aberrantly acquired events, doublets, debris, and beads. We then gated cell populations based of their expressions of CD3, CD4, CD8, CD19, CD56, CD14 and CD16. Marker expression dynamics were examined across the full spectrum of cells in the dataset, allowing for the determination of positive staining on individual cells against general background staining.

- [x] Tick this box to confirm that a figure exemplifying the gating strategy is provided in the Supplementary Information.
